# Attention-deficit/hyperactivity disorder as a risk factor for dementia and mild cognitive impairment: A population-based register study

**DOI:** 10.1192/j.eurpsy.2021.2261

**Published:** 2021-12-20

**Authors:** Maja Dobrosavljevic, Le Zhang, Miguel Garcia-Argibay, Ebba Du Rietz, Henrik Andershed, Zheng Chang, Stephen Faraone, Henrik Larsson

**Affiliations:** 1 School of Medical Sciences, Örebro University, Örebro, Sweden; 2 Department of Medical Epidemiology and Biostatistics, Karolinska Institutet, Stockholm, Sweden; 3 School of Law, Psychology and Social Work, Örebro University, Örebro, Sweden; 4 Departments of Psychiatry and of Neuroscience and Physiology, SUNY Upstate Medical University, Syracuse, New York, USA

**Keywords:** Attention-deficit/hyperactivity disorder, dementia, mild cognitive impairment, population-based study

## Abstract

**Background:**

Previous research has indicated that attention-deficit/hyperactivity disorder (ADHD) is associated with an increased risk for dementia, but studies are scarce and inconclusive. We aimed to investigate the association between ADHD, and dementia and mild cognitive impairment (MCI). Additionally, we aimed to investigate the impact of comorbid conditions, educational attainment, head injuries, other developmental disorders, and sex on the association.

**Methods:**

The study population consisted of 3,591,689 individuals born between 1932 and 1963, identified from Swedish population-based registers. Cases of ADHD, dementia and MCI were defined according to ICD diagnostic codes and ATC codes for medication prescriptions. A Cox proportional hazards model was used to test the associations between ADHD, and dementia and MCI.

**Results:**

Individuals with ADHD had an increased risk for dementia and MCI. After adjusting for sex and birth year, a hazard ratio (HR) was 2.92 (95% confidence interval 2.40–3.57) for dementia, and 6.21 (5.25–7.35) for MCI. Additional adjustment for psychiatric disorders (depression, anxiety, substance use disorder, and bipolar disorder) substantially attenuated the associations, HR = 1.62 (1.32–1.98) for dementia, and 2.54 (2.14–3.01) for MCI. Common metabolic disorders (hypertension, type 2 diabetes, and obesity), sleep disorders, head injuries, educational attainment, and other developmental disorders, had a limited impact on the association. The association between ADHD and dementia was stronger in men.

**Conclusions:**

ADHD is a potential risk factor for dementia and MCI, although the risk significantly attenuates after controlling for psychiatric disorders. Further research is needed to confirm these findings and to explore underlying mechanisms of the associations.

## Introduction

Attention-deficit/hyperactivity disorder (ADHD) is a common, childhood-onset neurodevelopmental disorder and an important risk factor for many psychiatric [1,2] and general medical disorders [2] across the life span. Symptoms of ADHD may persist to an older age in a substantial number of individuals with ADHD [3]. However, the extent to which individuals with ADHD are at increased risk for dementia and mild cognitive impairment (MCI) is unclear. Dementia is characterized by a significant decline in cognition, behavior and in the ability to perform everyday activities, whereas MCI is defined by the presence of impairment in one or more cognitive domains without affecting functional independence and without fulfilling the diagnostic criteria for dementia [4].

Studies in humans [5–8] and animal models of ADHD [9-10] have indicated an association between antecedent symptoms of ADHD and cognitive deficits in later life, but available literature is limited and conflicting. Three studies, including a population-based study from Taiwan [5], a U.S. hospitalization discharge, cohort study [6], and a case-control study from Argentina [7], found an increased risk for dementia in people with ADHD. In contrast, a cross-sectional study from the United States [8] did not find a significant association between childhood symptoms of ADHD and dementia/MCI in later life. Limitations of these studies were the use of inpatient-care data that cover more severe cases of ADHD and dementia [6], while most patients with ADHD are treated in outpatient care, and over-representation of males and individuals aged 18–54 years old in the study population [5]. The included individuals [5] might not represent the population at risk, as it has been reported that dementia is more prevalent in women and the risk of developing dementia before age 50 is low [11]. Other limitations were the retrospective assessment of childhood ADHD symptoms in older adults with and without cognitive impairment [7-8], allowing for recall bias and misclassification.

Additionally, it has been proposed [12] that ADHD might lead to development of cumulative health-compromising factors along the lifespan, which in turn present risk factors for developing MCI and dementia in later life. Low educational attainment, common metabolic disorders or metabolic syndrome (i.e., hypertension, type 2 diabetes [T2D] and obesity), sleep disorders, head injuries, and psychiatric disorders (i.e., depression, anxiety, substance use disorder [SUD], and bipolar disorder), are associated with ADHD [1,2], and well-established risk factors for dementia [13–16]. Unfortunately, only a few studies have explored the potential impact of these factors on the association between ADHD and dementia, with inconsistent findings. One study reported an increased risk for dementia in people with ADHD even after adjusting for socioeconomic status, general medical and psychiatric comorbidities [5]. In contrast, another study [6] found that the association between ADHD and dementia was no longer significant after controlling for metabolic dysregulation.

The current study aimed to extend previous research [5–8,12] by utilizing large-scale population data from national registers in Sweden, and a retrospective cohort design to investigate the association of ADHD with dementia and MCI. We additionally aimed to investigate to what extent these associations are affected by educational attainment, comorbid metabolic disorders (i.e., hypertension, T2D, and obesity), sleep disorders, head injuries, and psychiatric disorders (i.e., depression, anxiety, SUD, and bipolar disorder). We also investigated whether a potentially increased risk for dementia is specific to ADHD, or shared with comorbid developmental disorders [17]. Finally, since prevalence rates of ADHD [1] and dementia [11] differ between men and women, we aimed to explore whether their associations differ by sex.

## Method

### Data sources

We used data from multiple Swedish population-based registers. All individuals registered in Sweden are assigned a unique personal identity number that enables a linkage of these registers [18]. The Total Population Register (TPR) includes all individuals in Sweden born since 1932, who were alive in 1963 and later. We used the following demographic data from the TPR: date of birth, sex, date of death, and migration [18]. The National Patient Register (NPR) covers all primary, and up to eight secondary diagnoses from inpatient hospital admissions since 1987, and information from the outpatient register since 2001 [19]. The Cause of Death Register (CDR) contains information of all deaths since 1952 [20]. All diagnoses in the NPR and CDR were classified according to the International Classification of Diseases (ICD) versions 7/8/9/10. The Prescribed Drug Register (PDR) covers data on all dispensed medication prescriptions since July 1, 2005 using the Anatomical Therapeutic Classification (ATC) system, with a date of prescription and dosage. Longitudinal integration database for health insurance and labour market studies register (LISA) covers information on educational attainment for individuals aged ≥ 16 since 1990 [21].

### Study population

**Figure 1.**
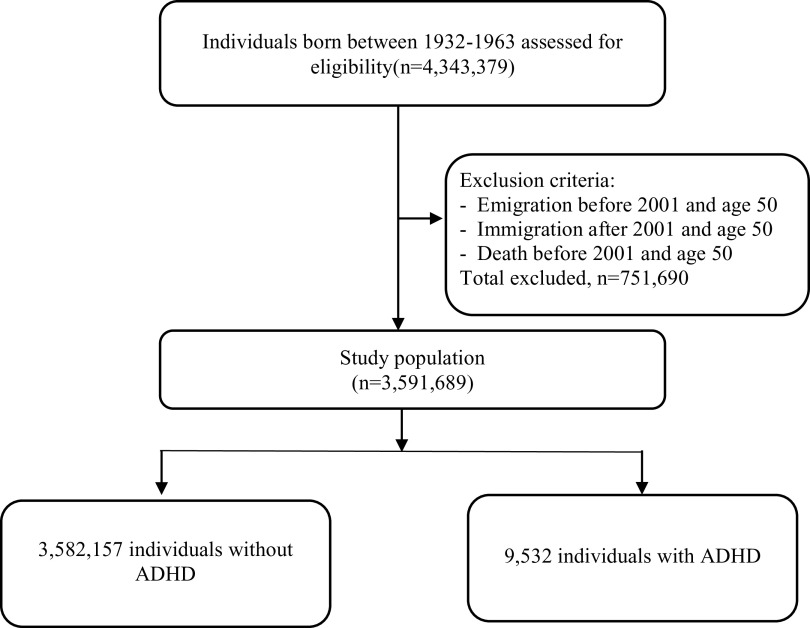
Flowchart of the study population selection process.

The total cohort consisted of 3,591,689 individuals born between 1932 and 1963, who were alive and resided in Sweden in 2001 (Figure 1). We excluded individuals who emigrated from Sweden and died before 2001, and before age 50, and those who immigrated to Sweden after 2001 and aged 50 and above. A diagnosis of ADHD has been mostly available from the outpatient care medical files recorded in the NPR since 2001. The beginning of the follow-up was set at age 50. The end of the follow-up was set at the date of a diagnosis of dementia/MCI, emigration, death, or December 31, 2013 (the last date with available data), whichever came first.

### Measures of ADHD

ADHD cases were defined as individuals who received an ICD-9/10 diagnosis from the NPR [22], or dispensed medication prescription for ADHD treatment according to the ATC codes from the PDR [23] (Supplementary Table S1), at any age. Medication prescriptions can be considered as valid indicators of ADHD diagnoses, and both ADHD diagnoses from the NPR and medication prescriptions from the PDR are provided exclusively by specialists in Sweden [22]. To additionally ensure that ADHD cases reflect clinically relevant diagnoses, we performed a sensitivity analysis by including only individuals with a confirmed diagnosis of ADHD from the NPR [24].

### Measures of dementia and MCI

We included a diagnosis of dementia or MCI recorded at age 50 or older to minimize the risk of misdiagnosis with conditions with similar clinical presentations (i.e., ADHD) when dementia/MCI is diagnosed at a younger age [25]. The following types of dementia were included: Alzheimer’s disease (AD), vascular dementia, and other dementias, with diagnostic codes according to the ICD-8/9/10 from the NPR and the CDR, or medication prescription for AD according to the ATC codes from the PDR (Supplementary Table S1) [26]. MCI was identified from the NPR in accordance with the ICD-10 (Supplementary Table S1) [4].

### Covariates

The following variables, associated with ADHD and/or dementia in previous research, were addressed as covariate sets: (a) sex [1,11] and birth year [11]; (b) educational attainment (highest level of education by age 50 with categories: compulsory education ≤9 years/upper secondary/postsecondary/postgraduate) [1,13]; (c) metabolic disorders: hypertension, T2D, and obesity [2,13]; (d) sleep disorders (organic and nonorganic) [2,15]; (e) head injuries [2,16]; (f) psychiatric disorders: depression, anxiety, SUD, and bipolar disorder; [1,2,13,14] (g) other developmental disorders (i.e., autism spectrum disorder, intellectual disability, motor disorders, and learning disorders) [17]. We extracted a first diagnosis of included disorders and head injuries, coded according to the ICD-8/9/10 from the NPR (Supplementary Table S1) and acquired at any age.

### Statistical analysis

We used a Cox proportional hazards model to test the association of ADHD with dementia/MCI, by comparing the rate of having these disorders between individuals with and without ADHD from the age of 50 years, with attained age as the underlying timescale. Since ADHD is defined according to the ICD-10 criteria by a childhood-onset of the symptoms [27], we considered individuals receiving a diagnosis of ADHD during the study period as exposed from the start of follow-up (i.e., from age 50). Hazard ratio (HR) was estimated with 95% confidence intervals (CI), with adjustment for sex and birth year, followed by separate adjustments for each covariate set.

We used Wilcoxon two-sample test to evaluate differences in median age of dementia/MCI diagnosis, and median age in 2013 between individuals with and without ADHD. When distribution of frequencies for covariates differed between groups with and without ADHD, we used logistic regression models to inspect associations between ADHD and the covariates, presented as odds ratios (OR) with 95 % CIs and adjusted for sex and birth year.

To investigate whether the case definition of ADHD based on a confirmed diagnosis would affect the results, we conducted a sensitivity analysis by excluding cases with ADHD medication prescription only.

Main and sensitivity analyses were presented collapsed across sex and stratified by sex. We used SAS version 9.4 (SAS Institute, Inc.) for data management and R version 3.6.1 for data analyses.

## Results

### Descriptive characteristics of the study population

The study population covered 3,591,689 individuals born between 1932 and 1963 (Figure 1). We identified 9,532 (0.3%) people diagnosed with ADHD diagnosis/medication prescription, among which 5,168 (54.2%) were male (Table 1). Median age of ADHD diagnosis/medication prescription was 52 years (IQR 48─57). By the end of the follow-up, 55,194 (1.5%) individuals developed dementia, and 23,507 (0.6%) developed MCI, with a median follow-up time of 14.13 years for both dementia and MCI. Median age at diagnosis for dementia and MCI was significantly lower in individuals diagnosed with ADHD compared to those without ADHD (Table 1; Wilcoxon Two-Sample Test: for dementia, *Z* = −9.02, *p* < 0.0001; for MCI, *Z* = −11.55, *p* < 0.0001). At the end of 2013 individuals with ADHD were younger than those without ADHD (Table 1; Wilcoxon Two-Sample Test: *Z* = −80.03, *p* < 0.0001). All covariates were significantly associated with ADHD (adjusted for sex and birth year, Table 2).

**Table 1.** Descriptive characteristics of the study population.

		Individuals without ADHD	Individuals with ADHD	Total *N* = 3,591,689
		*N* = 3,582,157	*N* = 9,532	
Male, *N* (%)		1,815,916 (50.7)	5,168 (54.2)	1,821,084 (50.7)
Female, *N* (%)		1,766,241 (49.3)	4,364 (45.8)	1,770,605 (49.3)
*N* on December 31, 2013		3,265,451	8,938	3,274,389
Median age in 2013 (IQR)		63 (56─70)	55 (52─60)	63 (56─70)
Dementia	All, *N* (%)	55,094 (1.5)	100 (1.0)	55,194 (1.5)
	Age at diagnosis median (IQR)	72 (67─76)	61.5 (56─70.5)	72 (67─76)
	Event rate per 10,000 person years	10.75 (10.66─10.84)	13.94 (11.30─17.01)	10.76 (10.67─10.85)
	Male, *N* (%)	27,126 (1.5)	64 (1.2)	27,190 (1.5)
	Age at diagnosis median (IQR)	72 (67─76)	61 (56─68)	72 (67─76)
	Event rate per 10,000 person years	10.57 (10.44─10.69)	17.13 (13.16─21.92)	10.58 (10.45─10.70)
	Female, *N* (%)	27,968 (1.6)	36 (0.8)	28,004 (1.6)
	Age at diagnosis median (IQR)	72 (68─76)	63.5 (56─73)	72 (68─76)
	Event rate per 10,000 person years	10.94 (10.81─11.07)	10.36 (7.18─14.48)	10.94 (10.81─11.07)
MCI	All, *N* (%)	23,365 (0.6)	142 (1.5)	23,507 (0.6)
	Age at diagnosis median (IQR)	69 (62─74)	59 (53─66)	69 (62─74)
	Event rate per 10,000 person years	4.51 (4.45─4.57)	19.89 (16.71─23.50)	4.53 (4.47─4.59)
	Male, *N* (%)	11,760 (0.6)	75 (1.4)	11,835 (0.6)
	Age at diagnosis median (IQR)	69 (62─74)	60 (53─65)	69 (62─74)
	Event rate per 10,000 person years	4.54 (4.46─4.63)	20.41 (16.06─25.59)	4.57 (4.48─4.65)
	Female, *N* (%)	11,605 (0.7)	67 (1.5)	11,672 (0.7)
	Age at diagnosis median (IQR)	69 (62─74)	58 (53─66)	69 (62─74)
	Event rate per 10,000 person years	4.48 (4.40─4.56)	19.30 (14.83─24.69)	4.50 (4.42─4.58)

Abbreviations: ADHD, Attention-deficit/hyperactivity disorder; MCI, Mild cognitive impairment.

**Table 2.** Descriptive characteristics of the study population with regard to educational attainment, metabolic and sleep disorders, head injuries, psychiatric disorders, and other developmental disorders, and the associations with ADHD, presented as odds ratios (OR) with 95% confidence intervals (CI), adjusted for sex and birth year.

	Without ADHD 3,582,157 *N* (%)	With ADHD 9,532 *N* (%)	Total 3,591,689 *N* (%)	OR (95% CI) adjusted for sex and birth year
Educational attainment^a^	3,369,548	9,492	3,379,040	–
Compulsory education ≤9 years^b^	947,636 (28.1)	2,109 (22.2)	949,745 (28.1)	1.00
Upper secondary	1,484,868 (44.1)	4,796 (50.5)	1,489,664 (44.1)	1.08 (1.03─1.12)
Postsecondary	902,231 (26.8)	2,510 (26.4)	904,741 (26.8)	0.78 (0.74─0.81)
Postgraduate	34,813 (1.0)	77 (0.8)	34,890 (1.03)	0.66 (0.53─0.83)
Hypertension	739,049 (20.6)	1712 (18.0)	740,806 (20.6)	1.64 (1.55─1.73)
Type 2 diabetes	261,175 (7.3)	646 (6.8)	261,821 (7.3)	1.62 (1.49─1.76)
Obesity	93,282 (2.6)	635 (6.7)	93,917 (2.6)	2.65 (2.44─2.87)
Sleep disorders	148,768 (4.1)	1572 (16.5)	150,340 (4.2)	4.70 (4.45─4.97)
Head injuries	109,831 (3.1)	952 (10.0)	110,783 (3.1)	3.83 (3.58─4.10)
Depression	193,946 (5.4)	4199 (44.0)	198,145 (5.5)	14.32 (13.74─14.92)
Anxiety	165,425 (4.6)	3963 (41.6)	169,388 (4.7)	14.09 (13.51─14.68)
Substance use disorder	197,001 (5.5)	3820 (40.1)	200,821 (5.6)	12.15 (11.65─12.68)
Bipolar disorder	27,010 (0.7)	1299 (13.7)	28,309 (0.8)	20.40 (19.20─21.67)
Other developmental disorders	14,334 (0.4)	1028 (10.8)	15,362 (0.4)	26.81 (25.05─28.69)

a Missing values for educational attainment: 212,649. These cases were deleted from the main analysis adjusted for educational attainment.

b Reference category is compulsory educational attainment.

### Main findings

We found an increased risk of having both dementia (HR = 2.92, 95% CI = 2.40─3.57) and MCI (HR = 6.21, 95% CI = 5.25─7.35) in individuals with ADHD compared with individuals without ADHD, adjusting for sex and birth year (Table 3). After separate adjustments of the analysis for each considered covariate set, we observed that educational attainment, metabolic disorders, sleep disorders, head injuries and other developmental disorders had a minimal impact on the associations of ADHD with dementia/MCI (Table 3). In contrast, the adjustment for psychiatric disorders significantly attenuated the observed associations with HR = 1.62, 95% CI = 1.32─1.98, for dementia, and HR = 2.54, 95% CI = 2.14─3.01, for MCI (Table 3).

**Table 3.** Association between ADHD and dementia and mild cognitive impairment (MCI) as hazard ratios (HR) with 95% confidence intervals (CI) adjusted for covariates, collapsed across sex, and stratified by sex.

	HR (95% CI) adjusted for sex and birth year	HR (95% CI) adjusted for sex and birth year						Full adjustment HR (95% CI)
		Educational attainment	Metabolic disorders	Sleep disorders	Head injury	Psychiatric disorders	Other developmental disorders	
Dementia	2.92 (2.40─3.57)	2.90 (2.37─3.54)	2.81 (2.30─3.43)	2.73 (2.23─3.33)	2.73 (2.23─3.33)	1.62 (1.32─1.98)	2.46 (2.01─3.00)	1.40 (1.15─1.72)
Male	3.70 (2.89─4.74)	3.65 (2.85─4.68)	3.51 (2.74─4.49)	3.50 (2.73─4.48)	3.48 (2.72─4.46)	1.92 (1.49─2.46)	3.11 (2.42─3.98)	1.68 (1.31─2.16)
Female	2.11 (1.51─2.96)	2.10 (1.50─2.94)	2.06 (1.47─2.88)	1.94 (1.38─2.71)	1.95 (1.40─2.74)	1.24 (0.88─1.73)	1.78 (1.27─2.50)	1.07 (0.76─1.50)
MCI	6.21 (5.25─7.35)	5.77 (4.87─6.82)	5.81 (4.91─6.87)	4.81 (4.06─5.69)	5.58 (4.72─6.60)	2.54 (2.14─3.01)	5.47 (4.62─6.48)	2.05 (1.72─2.43)
Male	6.58 (5.24─8.27)	6.06 (4.82─7.61)	6.07 (4.84─7.63)	5.34 (4.25─6.71)	5.86 (4.66─7.36)	2.68 (2.12─3.38)	5.89 (4.68─7.42)	2.19 (1.74─2.77)
Female	5.86 (4.57─7.52)	5.48 (4.28─7.03)	5.55 (4.33─7.12)	4.27 (3.33─5.48)	5.34 (4.16─6.84)	2.41 (1.87─3.10)	5.09 (3.96─6.55)	1.92 (1.49─2.47)

Analyses stratified by sex were not additionally adjusted for sex.

The association between ADHD and dementia across all levels of adjustments was stronger in men compared to women (Table 3), with a significant ADHD by sex interaction coefficient, 0.58, 95% CI = 0.38─0.88, *p* = 0.01, meaning that the risk of having dementia in women with ADHD was 42% lower than in men with ADHD. An event rate per 10,000 person years for dementia was lower in women (10.36, 95% CI = 7.18─14.48) than men (17.13, 95% CI = 13.16─21.92) with ADHD, while the event rates for men and women without ADHD were equal (Table 1). On the other hand, the risk of having MCI was not significantly different between men and women with ADHD (ADHD by sex interaction coefficient of 0.97, 95% CI = 0.69─1.35, *p* = 0.84).

### Sensitivity analyses

We performed a sensitivity analysis to investigate whether the results were affected by the case identification for ADHD that revealed the same pattern of results across adjustments for all included covariate sets with overlapping confidence intervals, but somewhat lower associations of ADHD with dementia/MCI compared to the main analysis (Supplementary Table S2). When case identification of ADHD was based on a diagnosis only, point estimates for dementia attenuated towards the null and became nonsignificant after adjusting for psychiatric disorders.

Since the median age of ADHD and MCI/dementia diagnoses were relatively close in time and to provide further confirmation of the clinical validity of ADHD diagnoses, we conducted two post hoc sensitivity analyses, adjusted for sex and birth year, based on more sctrict case defitions of ADHD by including (a) only cases with at least two confirmed diagnoses of ADHD and (b) only cases with a primary diagnosis of ADHD. Both post hoc sensitivity analyses yielded results consistent with the main analysis (Supplementary Material, p. 4).

## Discussion

In this Swedish population-based register study, we found that ADHD was associated with an increased risk for both dementia and MCI. Psychiatric disorders substantially attenuated the associations of ADHD with dementia and MCI. Additionally, we demonstrated that the association between ADHD and dementia was stronger in men than in women.

Our findings support previous studies that identified an increased risk for dementia in people with antecedent ADHD [5–7]. One study, which did not find an association [8], applied a restrospective assessment of childhood ADHD sympoms in a small geriatric sample, while the current study used population-based, large scale data and ICD-diagnoses of ADHD. Furthermore, a recently published study that used Swedish health registers [28] identified an association between ADHD and Alzheimer’s disease and any dementia across generations.

Additionally, findings of the current study support a hypothesis that cumulative health-compromising factors of ADHD may affect the association between ADHD and dementia [12]. Specifically, we found that psychiatric disorders (i.e., anxiety, depression, SUD, and bipolar disorder), highly comorbid with ADHD [1,2], may partially explain the associations. Midlife psychiatric disorders present significant risk factors for dementia, independently from physical comorbidities [13,14]. It has been proposed that ADHD symptoms could affect cognitive functioning in older age indirectly through depressive symptoms. [29,30] Plausible underlying mechanisms, such as increased oxidative stress and inflammation, linked to both psychiatric disorders and dementia [31], should be considered in future research. Future longitudinal studies are required to elucidate the timeline between the onset of ADHD, other psychiatric disorders, and dementia.

Our finding that educational attainment had a limited influence on the association between ADHD and dementia needs to be interpreted carefully. Individuals with ADHD were more likely to complete secondary education, but less likely to complete postsecondary/postgraduate education compared to those without ADHD. This is somewhat inconsistent with previous studies that have identified an association between ADHD and lower educational levels [1]. A potential explanation is that older adults with low education remain undiagnosed for ADHD in Sweden. Additionally, individuals with more severe ADHD and low education may not live to an older age, due to increased mortality rates associated with ADHD [32]. Future studies need to explore whether our findings generalize to other settings.

We found an increased risk for common metabolic (i.e., hypertension, T2D, obesity) and sleep disorders, and head injuries in individuals with ADHD. Still, these covariates only minimally affected the associations of ADHD with dementia/MCI. One study [6] found that metabolic dysregulation mediated the association between ADHD and dementia. However, metabolic disorders might have been underdiagnosed in our cohort, which could have attenuated their effect on the association between ADHD and dementia/MCI. Individuals with ADHD have low rates of seeking medical treatment, despite having an increased risk for overall health problems [1]. Additionally, individuals with ADHD in our cohort were significantly younger at the end of the follow-up period and had lower crude prevalence estimates for T2D and hypertension, than those without ADHD. Since the rates of T2D and hypertension increase with age [2], the group with ADHD in this population might have not developed these disorders to the full extent due to younger age.

Further, our findings suggest that the association between ADHD and dementia/MCI may be independent from other developmental disorders. We were unable to address other early-life and neurobiological/genetic explanations to the observed associations [12]. Early-life adverse factors, both prenatal (i.e., fetal stress and low birth weight) and postnatal (i.e., psychosocial adversity) have been recognized as risk factors for ADHD [33], as well as MCI/dementia [34]. A large genome-wide, cross-disorder study failed to find statistically significant genetic correlations between ADHD and dementia [35], but the findings of this study are inconclusive as only the overlap between common genetic variants was explored, explaining a small proportion of the heritability. Further, the current study design did not allow us to differentiate between the effect of ADHD and ADHD medication on dementia/MCI. Future research is needed to investigate early-life and neurobiological/genetic mechanisms underlying the link between ADHD and dementia, and a potential role of medication.

The association between ADHD and dementia was somewhat stronger in men, while previous studies did not address the effect of sex on the association [6–8], or they found a stronger association in women [5]. We also found higher rates of dementia in men than women with ADHD. It is possible that dementia is underdiagnosed in women with ADHD. Alternatively, as ADHD is potentially underdiagnosed in females [1], it is plausible that preceding symptoms of ADHD were not recognized in some women who developed dementia.

The current study identified a prevalence of clinically diagnosed ADHD of 0.3%. This is notably lower compared to a pooled prevalence estimate based on validated scales of 2.5% in adults [36] and 2.2% in older adults [3], but consistent with the pooled prevalence of 0.2% based on clinically diagnosed ADHD in adults older than 50 [3]. Although the prevalence of ADHD declines with age [36], it may be underdiagnosed in older age due to (a) ADHD being misdiagnosed as age-related cognitive decline or other psychiatric conditions [25], (b) inadequate diagnostic criteria for this age group [36], and (c) only the most severe forms of ADHD being diagnosed, and/or (4) premature mortality associated with ADHD [37]. Further, a study from the Netherlands reported a population weighted prevalence of ADHD of 2.8% in older adults based on an ADHD diagnostic interview [38], and may additionally indicate a substantial underestimation of the prevalence in the current study. Future studies using validated instruments for assessment of ADHD in large community samples of older adults are therefore needed to replicate the current findings.

## Limitations

Some limitations of the current study should be addressed when interpreting its findings. The risk for MCI in people with ADHD was larger than the risk for dementia, and the median age of diagnosis for ADHD and MCI/dementia were close in time. In older adults, symptoms of ADHD, such as difficulty to organize activities, inability to sustain attention, memory problems, behavioral and psychiatric symptoms (e.g., sleep disturbances, anxiety, and depression) may resemble prodromal dementia/MCI [25]. Additionally, other psychiatric conditions with similar clinical presentation may be misdiagnosed as ADHD, or vice versa [25]. The question of misdiagnosis could not be fully addressed due to limited information on dementia/MCI diagnoses provided in the registers. We partially addressed this issue by re-running the analyses including only cases with at least two established diagnoses and only cases with a primary diagnosis of ADHD, which confirmed our main findings. Future validation studies of MCI and adult ADHD classification criteria in Swedish registries are warranted.

We could not investigate separate associations between ADHD and dementia subtypes (i.e., AD, vascular dementia, dementia with Lewy bodies, frontotemporal dementia) due to a small number of individuals with ADHD diagnosed with each subtype. It is plausible that different subtypes of dementia, having distinct etiological pathways, are differentially associated with ADHD and comorbid disorders. Additionally, ADHD and some subtypes of dementia might only share similar clinical presentation; however, underlying mechanisms could be distinct and unrelated [12]. For instance, previous research has found no or very little evidence for an association between AD and ADHD [5–8]. Future studies should focus on building a more comprehensive model of possible associations between ADHD and different subtypes of dementia, and on investigating the role of specific comorbid disorders for each association.

We did not cover behavioral outcomes (i.e., smoking, diet, exercise, and treatment-seeking behaviors) of ADHD that could affect the association between ADHD and dementia [12], since this information was not available in the registers. Future studies should aim to include relevant behavioral variables.

The median age of the study population at the end of the follow-up was 63 years, while the risk for dementia substantially increases after the age of 65 [11], thus, we might have mostly captured early-onset cases of dementia. Further follow-up of these individuals to a more advanced older age would be necessary to explore the association with later-onset dementia. The use of a relatively young cohort may explain our finding of similar event rates of dementia for men and women without ADHD, which is inconsistent with reported higher rates of dementia in women in the general population, since potential sex differences could be present after age 90 [11]. Further, the number of dementia cases were almost twice the number of MCI cases and only 9,908 individuals had both MCI and dementia. It is plausible that some individuals tend to seek medical help only when they experience more severe cognitive symptoms due to social stigma [39]. Because of the younger age of the cohort, the remaining individuals with MCI might develop dementia in the future.

## Conclusions

In conclusion, the present study suggests that there is an increased risk for dementia and MCI in individuals with ADHD, with the association between ADHD and dementia being stronger in men than in women. These associations substantially attenuate after controlling for comorbid psychiatric disorders, while common metabolic disorders and sleep disorders, head injuries, educational attainment, and other developmental disorders, have a limited impact. Since this is a notably understudied topic, more research is of crucial importance to confirm our findings and investigate underlying mechanisms. Specifically, future neurobiological and epidemiological studies are needed, which should cover different subtypes of dementia, behavioral outcomes of ADHD, and a broader range of socioeconomic variables and comorbid health conditions.

## Data Availability

The Public Access to Information and Secrecy Act in Sweden prohibits individual level data to be publicly available. Researchers who are interested in replicating this study can apply for individual level data at Statistics Sweden: www.scb.se/en/services/guidance-for-researchers-and-universities/.

## Supplementary Materials

To view supplementary material for this article, please visit http://doi.org/10.1192/j.eurpsy.2021.2261. click here to view supplementary material

## References

[1] Fayyad J, Sampson NA, Hwang I, Adamowski T, Aguilar-Gaxiola S, Al-Hamzawi A, et al. The descriptive epidemiology of DSM-IV adult ADHD in the world health organization world mental health surveys. Atten Defic Hyperact Disord. 2017;9:47–65. doi:10.1007/s12402-016-0208-3. 27866355 PMC5325787

[2] Nigg JT. Attention-deficit/hyperactivity disorder and adverse health outcomes. Clin Psychol Rev. 2013;33:215–28. doi:10.1016/j.cpr.2012.11.005. 23298633 PMC4322430

[3] Dobrosavljevic M, Solares C, Cortese S, Andershed H, Larsson H. Prevalence of attention-deficit/hyperactivity disorder in older adults: a systematic review and meta-analysis. Neurosci Biobehav Rev. 2020;118:282–9. doi:10.1016/j.neubiorev.2020.07.042. 32798966

[4] Winblad B, Palmer K, Kivipelto M, Jelic V, Fratiglioni L, Wahlund LO, Nordberg A, et al. Mild cognitive impairment–beyond controversies, towards a consensus: report of the International Working Group on Mild Cognitive Impairment. J Intern Med. 2004;256:240–6. doi:10.1111/j.1365-2796.2004.01380.x. 15324367

[5] Tzeng NS, Chung CH, Lin FH, Yeh CB, Huang SY, et al. Risk of dementia in adults with ADHD: a nationwide, population-based cohort study in Taiwan. J Atten Disord. 2019;23:995–1006. doi:10.1177/1087054717714057. 28629260

[6] Fluegge K, Fluegge K. Antecedent ADHD, dementia, and metabolic dysregulation: a U.S. based cohort analysis. Neurochem Int. 2018;112:255–8. doi:10.1016/j.neuint.2017.08.005. 28811268

[7] Golimstok A, Rojas JI, Romano M, Zurru MC, Doctorovich D, Cristiano E. Previous adult attention-deficit and hyperactivity disorder symptoms and risk of dementia with Lewy bodies: a case-control study. Eur J Neurol. 2011;18:78–84. doi:10.1111/j.1468-1331.2010.03064.x. 20491888

[8] Ivanchak N, Abner EL, Carr SA, Freeman SJ, Seybert A, Ranseen J, et al. Attention-deficit/hyperactivity disorder in childhood is associated with cognitive test profiles in the geriatric population but not with mild cognitive impairment or Alzheimer’s disease. J Aging Res. 2011;2011:729801. doi:10.4061%2F2011%2F729801. 10.4061/2011/729801 PMC3142705 21822493

[9] Zhang Q, Du G, John V, Kapahi P, Bredesen DE. Alzheimer’s model develops early ADHD syndrome. J Neurol Neurophysiol. 2015;6:1–6. 26753104 PMC4704098

[10] Dellu-Hagedorn F, Trunet S, Simon H. Impulsivity in youth predicts early age-related cognitive deficits in rats. Neurobiol Aging. 2004;25:525–37. doi:doi/10.1016/j.neurobiolaging.2003.06.006. 15013574 10.1016/j.neurobiolaging.2003.06.006

[11] Ruitenberg A, Ott A, van Swieten JC, Hofman A, Breteler MM. Incidence of dementia: does gender make a difference? Neurobiol Aging. 2001;22:575–80. doi:10.1016/s0197-4580(01)00231-7. 11445258

[12] Callahan BL, Bierstone D, Stuss DT, Black SE. Adult ADHD: risk factor for dementia or phenotypic mimic? Front Aging Neurosci. 2017;9:260. doi:10.3389/fnagi.2017.00260. 28824421 PMC5540971

[13] Norton S, Matthews FE, Barnes DE, Yaffe K, Brayne C. Potential for primary prevention of Alzheimer’s disease: an analysis of population-based data. Lancet Neurol. 2014;13:788–94. doi:10.1016/s1474-4422(14)70136-x 25030513

[14] Zilkens R, G Bruce D, Duke J, Spilsbury K, B Semmens J. Severe psychiatric disorders in mid-life and risk of dementia in late-life (age 65–84 years): a population based case-control study. Curr Alzheimer Res. 2014;11:681–93. doi:10.2174/1567205011666140812115004. 25115541 PMC4153082

[15] Shi L, Chen SJ, Ma MY, Bao YP, Han Y, Wang YM, et al. Sleep disturbances increase the risk of dementia: a systematic review and meta-analysis. Sleep Med Rev. 2018; 40:4–16. doi:10.1016/j.smrv.2017.06.010. 28890168

[16] Li Y, Li Y, Li X, Zhang S, Zhao J, Zhu X, et al. Head injury as a risk factor for dementia and Alzheimer’s disease: a systematic review and meta-analysis of 32 observational studies. PloS One. 2017;12:e0169650. doi:10.1371%2Fjournal.pone.0169650. 28068405 10.1371/journal.pone.0169650 PMC5221805

[17] Selinus EN, Molero Y, Lichtenstein P, et al. Childhood symptoms of ADHD overrule comorbidity in relation to psychosocial outcome at age 15: a longitudinal study. PloS One. 2015;10:e0137475. doi:10.1371/journal.pone.0137475. PMC4567137 26360378

[18] Ludvigsson JF, Almqvist C, Bonamy AK, Ljung R, Michaëlsson K, Neovius M, et al. Registers of the Swedish total population and their use in medical research. Eur J Epidemiol. 2016;31:125–136. doi:10.1007/s10654-016-0117-y. 26769609

[19] Ludvigsson JF, Andersson E, Ekbom A, Feychting M, Kim JL, Reuterwall C, et al. External review and validation of the Swedish national inpatient register. BMC Public Health. 2011;11:450. doi:10.1186/1471-2458-11-450. 21658213 PMC3142234

[20] Brooke HL, Talbäck M, Hörnblad J, Johansson LA, Ludvigsson JF, Druid H, et al. The Swedish cause of death register. Eur J Epidemiol. 2017;32:765–73. doi:10.1007/s10654-017-0316-1. 28983736 PMC5662659

[21] Ludvigsson JF, Svedberg P, Olén O, Bruze G, Neovius M. The longitudinal integrated database for health insurance and labour market studies (LISA) and its use in medical research. Eur J Epidemiol. 2019;34:423–37. doi:10.1007/s10654-019-00511-8. 30929112 PMC6451717

[22] Larsson H, Rydén E, Boman M, Långström N, Lichtenstein P, Landén M. Risk of bipolar disorder and schizophrenia in relatives of people with attention-deficit hyperactivity disorder. Br J Psychiatry. 2013;203:103–6. doi:10.1192/bjp.bp.112.120808. 23703314 PMC3730113

[23] Zetterqvist J, Asherson P, Halldner L, Långström N, Larsson H. Stimulant and non‐stimulant attention deficit/hyperactivity disorder drug use: total population study of trends and discontinuation patterns 2006–2009. Acta Psychiatr Scand. 2013;128:70–7. doi:10.1111/acps.12004. 22943458

[24] Ghirardi L, Brikell I, Kuja-Halkola R, Freitag CM, Franke B, Asherson P, et al. The familial co-aggregation of ASD and ADHD: a register-based cohort study. Mol Psychiatry. 2018;23:257–62. doi:10.1038/mp.2017.17. 28242872 PMC5794881

[25] Pollak J. Distinguishing between adult ADHD and mild cognitive impairment. Curr Psychiatr. 2012;11:48.

[26] Eriksson LI, Lundholm C, Narasimhalu K, Sandin R, Jin YP, Gatz M, et al. Hospitalization, surgery, and incident dementia. Alzheimers Dement. 2019;15:534–42. doi:10.1016/j.jalz.2018.12.005 30777379 PMC6461518

[27] World Health Organization. The ICD-10 classification of mental and behavioural disorders: clinical descriptions and diagnostic guidelines. Geneva: World Health Organization; 1992.

[28] Zhang L, Du Rietz E, Kuja‐Halkola R, Dobrosavljevic M, Johnell K, Pedersen NL, et al. Attention‐deficit/hyperactivity disorder and Alzheimer’s disease and any dementia: a multi‐generation cohort study in Sweden. Alzheimer’s Dement. 2021;9:1–9. doi:10.1002/alz.12462. 34498801

[29] Das D, Cherbuin N, Easteal S, Anstey KJ. Attention Deficit/Hyperactivity Disorder symptoms and cognitive abilities in the late-life cohort of the PATH through life study. PloS One. 2014;9:e86552. doi:10.1371/journal.pone.0086552. 24489743 PMC3904910

[30] Semeijn E, Korten N, Comijs H, Michielsen M, Deeg D, Beekman A, et al. No lower cognitive functioning in older adults with attention-deficit/hyperactivity disorder. Int Psychogeriatr. 2015;27:1467–76. doi:10.1017/s1041610215000010. 25655491

[31] Kim HK, Nunes PV, Oliveira KC, Young LT, Lafer B. Neuropathological relationship between major depression and dementia: a hypothetical model and review. Prog Neuropsychopharmacol Biol Psychiatry. 2016;67:51–7. doi:10.1016/j.pnpbp.2016.01.008. 26780170

[32] Dalsgaard S, Østergaard SD, Leckman JF, Mortensen PB, Pedersen MG. Mortality in children, adolescents, and adults with attention deficit hyperactivity disorder: a nationwide cohort study. Lancet. 2015;385:2190–6. doi:10.1016/s0140-6736(14)61684-6. 25726514

[33] Banerjee TD, Middleton F, Faraone SV. Environmental risk factors for attention‐deficit hyperactivity disorder. Acta Paediatr. 2007;96:1269–74. doi:10.1111/j.1651-2227.2007.00430.x. 17718779

[34] Seifan A, Schelke M, Obeng-Aduasare Y, Isaacson R. Early life epidemiology of Alzheimer’s disease—a critical review. Neuroepidemiology. 2015;45:237–54. doi:10.1159/000439568. 26501691 PMC4679687

[35] Anttila V, Bulik-Sullivan B, Finucane HK, Walters RK, Bras J, Duncan L, et al. Analysis of shared heritability in common disorders of the brain. Science. 2018;360:eaap8757. doi:10.1126/science.aap8757. 29930110 PMC6097237

[36] Simon V, Czobor P, Balint S, Meszaros A, Bitter I. Prevalence and correlates of adult attention-deficit hyperactivity disorder: meta-analysis. Br J Psychiatry. 2009;194:204–11. doi:10.1192/bjp.bp.107.048827. 19252145

[37] Sun S, Kuja-Halkola R, Faraone SV, D’Onofrio BM, Dalsgaard S, Chang Z, et al. Association of psychiatric comorbidity with the risk of premature death among children and adults with attention-deficit/hyperactivity disorder. JAMA Psychiatry. 2019;76:1141–9. doi:10.1001/jamapsychiatry.2019.1944. 31389973 PMC6686979

[38] Michielsen M, Semeijn E, Comijs HC, van de Ven, P, Beekman, AT, Deeg, DJ, et al. Prevalence of attention-deficit hyperactivity disorder in older adults in the Netherlands. Br J Psychiatry. 2012;201:298–305. doi:10.1192/bjp.bp.111.101196. 22878132

[39] Phillipson L, Magee C, Jones S, Reis S, Skaldzien E. Dementia attitudes and help-seeking intentions: an investigation of responses to two scenarios of an experience of the early signs of dementia. Aging Ment Health. 2015;19:968–77. doi:10.1080/13607863.2014.995588. 25554920

