# Supplementary material for: Attention-deficit/hyperactivity disorder as a risk factor for dementia and mild cognitive impairment: A population-based register study
Source: Eur Psychiatry. 2021 Dec 20;65(1):e3. doi: 10.1192/j.eurpsy.2021.2261 (PMC8792867; doi:10.1192/j.eurpsy.2021.2261)
Supplement: Supplementary file 1 [file S0924933821022616sup001.docx]

**Supporting information**

**Table 1.** ICD and ATC codes used for case identification

|  | | **ICD-8** | **ICD-9** | **ICD-10** | **ATC** |
| --- | --- | --- | --- | --- | --- |
| **Years** | | 1969-1986 | 1987-1996 | 1997-2012 | 2005-2014 |
| **ADHD** | | - | 314 | F90 | N06BA01, N06BA02, N06BA09, 06BA12, C02AC02 |
| **Alzheimer (AD)** | | 290 | 290A/B/X, 331A | F00, F03, G30 | N06DA02-N06DA04, N06DX01 |
| **Vascular dementia** | | 293.0-293.1 | 290E | F01 | - |
| **Other dementia** | | - | 294B, 290W, 331B/C/X | F02, F02.1, F02.2, F02.3, F02.4, F02.8, F05.1, G31.1, G31.8 | - |
| **Mild cognitive impairment** | | - | - | F06.7 | - |
| **Hypertension** | | 400 – 404 | 401 – 405 | I10 – I13, I15 | - |
| **Type 2 diabetes mellitus** | | - | - | E11 | - |
| **Obesity** | | 277.99 | 278A, 278B | E65-E66 | - |
| **Sleep disorders** | | 347, 780.60 | 347, 780F | G47.0/1/2/3/4/8/9, F51 | - |
| **Head Injuries** | | 800, 801, 803, 850‐854 | 800, 801, 803, 850‐854 | S020, S021, S027‐S029, S060‐S071 | - |
| **Depression** | | 296.00, 300.40 | 296B, 300E, 311 | F32, F33 | - |
| **Anxiety** | | 300 (except 300.4) | 300 (except 300E) | F40, F41, F42, F44, F45, F48 | - |
| **Substance use disorder** | | 303, 304 | 303, 304, 305 | F10 – F19 | - |
| **Bipolar disorder** | | 296.10, 296.30, 296.88 | 296C/D/E/W | F31 | - |
| **Other developmental**  **disorders** | **Autism spectrum disorder** | - | 299A | F84.0, F84.1, F84.5, F84.8, F84.9 | - |
|  | **Developmental disorders of speech/language & Learning disorders** | - | 315A, 315B, 315D, 315W | F80, F81, F83 | - |
|  | **Intellectual disability** | 310–315 | 317-319 | F70-73, F78-79 | - |
|  | **Motor disorders** | - | 307D, 315E, 307C | F98.4, F82, F95 | - |

**Table 2.** Association between ADHD, and dementia and MCI, with ADHD cases identification based on a diagnosis only (hazard ratios with 95% confidence intervals)

|  | HR (95% CI) adjusted for sex and birth year | HR (95% CI) adjusted for sex and birth year, and: | | | | | | Full adjustment  HR (95% CI) |
| --- | --- | --- | --- | --- | --- | --- | --- | --- |
|  |  | Educational attainment | Metabolic disorders | Sleep disorders | Head injury | Psychiatric disorders | Other developmental disorders |  |
| **Dementia** | **2.93 (2.15-4.00)** | **2.81 (2.06-3.84)** | **2.86 (2.10-3.90)** | **2.87 (2.10-3.91)** | **2.67 (1.96-3.64)** | **1.19 (0.87-1.63)** | **2.06 (1.51-2.82)** | **0.98 (0.72-1.34)** |
| Male | 4.06 (2.87- 5.74) | 3.84 (2.72-5.44) | 3.88 (2.74-5.49) | 3.96 (2.80-5.61) | 3.64 (2.57-5.15) | 1.56 (1.10-2.21) | 2.95 (2.08-4.19) | 1.29 (0.91-1.83) |
| Female | 1.39 (0.70-2.79) | 1.36 (0.68-2.73) | 1.40 (0.70-2.81) | 1.36 (0.68-2.73) | 1.30 (0.65-2.60) | 0.60 (0.30-1.20) | 0.94 (0.47-1.89) | 0.50 (0.25-1.00) |
| **MCI** | **6.39 (5.11-8.00)** | **6.03 (4.81-7.54)** | **6.05 (4.83- 7.57)** | **5.65 (4.51- 7.08)** | **5.55 (4.43-6.95)** | **2.04 (1.63-2.56)** | **5.19 (4.13- 6.52)** | **1.71 (1.36-2.15)** |
| Male | 7.30 (5.49-9.70) | 6.82 (5.13-9.06) | 6.81 (5.12-9.06) | 6.50 (4.88-8.64) | 6.18 (4.65-8.23) | 2.38(1.78-3.17) | 6.15 (4.61-8.22) | 2.00 (1.49-2.68) |
| Female | 5.37 (3.72-7.74) | 5.09 (3.53- 7.34) | 5.15 (3.57-7.42) | 4.69 (3.25-6.76) | 4.83 (3.35-6.96) | 1.69 (1.17- 2.44) | 4.20 (2.90- 6.07) | 1.42 (0.98-2.05) |

Note: Mild cognitive impairment: MCI, Attention-deficit/hyperactivity disorder: ADHD; Analyses stratified by sex were not additionally adjusted for sex; Number of cases with ADHD diagnosis, N=6753; Number of cases with both ADHD and dementia, N= 43 (male N=33); Number of cases with both ADHD and MCI, N= 80 (male N=48).

Number of cases with ADHD medication prescription among those diagnosed with ADHD (6753), N=5022 (74.37%).

(Among those who were prescribed with an ADHD medication (N=7801) in the total population of ADHD individuals (N=9532), 2779 (35.62%) individuals did not receive an ADHD diagnosis.)

|  |  |
| --- | --- |

**Post hoc sensitivity analyses**

- **Association between ADHD diagnosed at least two times, and dementia and MCI**

Number of cases with at least two ADHD diagnoses, N= 5436; Number of cases with both ADHD and dementia, N=27; Number of cases with both ADHD and MCI, N=59, This analysis was done with basic adjustment for sex and birth year only, due to a small number of cases.

Association between ADHD and dementia: HR=2.54, 95% CI 1.70 to 3.80, male: HR= 3.41, 2.15 to 5.42; female: HR=1.45, 0.65 to 3.22.

Association between ADHD and MCI: HR=6.19, 95% CI 4.76 to 8.06, male: HR=7.04, 5.02 to 9.87, female: HR=5.30, 3.48 to 8.06.

- **Association between ADHD as a primary and any secondary diagnosis, and dementia and MCI**

Number of cases with ADHD as a primary/main diagnosis, N=4452, number of cases with ADHD and dementia, N=19 (0.43%), number of cases with both ADHD and MCI, N=36 (0.81%).

Association between ADHD as a primary diagnosis and dementia, HR=2.08, 95% CI 1.31 to 3.30 (adjusted for sex and birth year).

Association between ADHD as a primary diagnosis and MCI, HR= 4.35, 95% CI 3.10 to 6.09 (adjusted for sex and birth year).

Number of cases with any secondary diagnosis, N=2301, number of cases with ADHD and dementia, N=24 (1.04%), and number of cases with ADHD and MCI, N=44 (1.91%).

Association between ADHD as any secondary diagnosis and dementia, HR=4.41, 95% CI 2.91-6.71 (adjusted for sex and birth year).

Association between ADHD as any secondary diagnosis and MCI, HR= 10.06, 95% CI 7.45 to 13.57 (adjusted for sex and birth year).
